# Respiratory diseases are positively associated with PM_2.5_ concentrations in different areas of Taiwan

**DOI:** 10.1371/journal.pone.0249694

**Published:** 2021-04-22

**Authors:** Feifei Wang, Tianyi Chen, Qian Chang, Yi-Wei Kao, Jian Li, Mingchih Chen, Yang Li, Ben-Chang Shia

**Affiliations:** 1 Center for Applied Statistics, Renmin University of China, Beijing, China; 2 School of Statistics, Renmin University of China, Beijing, China; 3 College of Management, Fu Jen Catholic University, Taipei, Taiwan; Universidade Federal do Rio Grande - FURG, BRAZIL

## Abstract

The health effects associated with fine particulate matter (PM_2.5_) have attracted considerable public attention in recent decades. It has been verified that PM_2.5_ can damage the respiratory and cardiovascular systems and cause various diseases. While the association between diseases and PM_2.5_ has been widely studied, this work aims to analyze the association between PM_2.5_ and hospital visit rates for respiratory diseases in Taiwan. To this end, a disease mapping model that considers spatial effects is applied to estimate the association. The results show that there is a positive association between hospital visit rates and the PM_2.5_ concentrations in the Taiwanese population in 2012 after controlling for other variables, such as smoking rates and the number of hospitals in each region. This finding indicates that control of PM_2.5_ could decrease hospital visit rates for respiratory diseases in Taiwan.

## Introduction

Every day, hundreds of millions of people worldwide suffer from various respiratory diseases. According to Feldman and Richards (2018) [1], lower respiratory infections alone are the fifth leading cause of death worldwide. After decades of research, scholars have identified certain causes of these diseases, such as genetic issues, infections, a nd smoking [2, 3]. In addition, air pollution has adverse health effects on the respiratory system and, thus, causes many respiratory diseases. In particular, patients of chronic respiratory diseases, such as chronic obstructive pulmonary disease (COPD) and the onset of asthma, are more vulnerable to air pollution [4]. Furthermore, 91% of the world’s population lives in places where the air quality fails to meet the World Health Organization (WHO) standards [5], which is a global environmental problem. Specifically, a major health-damaging component of air pollution is fine particulate matter with a diameter less than 2.5 *μm* (PM_2.5_). These particles can penetrate the lungs and bloodstream unfiltered, causing respiratory diseases [6].

Studies on the association between PM_2.5_ and respiratory diseases have attracted considerable attention. An early study found that lung cancer mortality could increase 8% for every 10 *μg*/*m*^3^ increase in PM_2.5_ [7]. Another study conducted in the United States found that respiratory deaths could increase by 1.68% for every 10 *μg*/*m*^3^ increase in 2-day averaged PM_2.5_ concentrations [8]. Similar conclusions are drawn in studies from Europe and Japan [9, 10].

However, studies on the association between PM_2.5_ and respiratory diseases are rare in Taiwan. Owing to the prevalence of PM_2.5_ in Taiwan and the consequent health risks, it is of great importance to conduct more research to explore such associations. Meanwhile, owing to infectiousness, many respiratory diseases show the characteristics of spatial aggregation. Therefore, spatial factors are often considered in public health studies [7, 11]. In this work, a coherent generative model [12] is applied to explore the relationship between PM_2.5_ and respiratory diseases in Taiwan. As shown by [12], this model can provide more accurate estimates and tighter credible intervals than previous methods. In this work, we applied this model to investigate the relationship between PM_2.5_ and hospital visit rates for respiratory diseases in Taiwan. By controlling smoking rates and the number of hospitals in each region, a significantly positive effect from PM_2.5_ concentrations on the hospital visit rates for respiratory diseases was found.

## Materials and methods

### Data sources

In this work, the research objective is to investigate the influence of PM_2.5_ on hospital visit rates for respiratory diseases in Taiwan. The data of hospital visits were collected for different diseases in 349 third-level administrative regions of Taiwan in 2012 from the National Health Insurance Research Database (NHIRD, https://nhird.nhri.org.tw/). Then, the hospital visit rates for respiratory diseases are defined as hospital visits for respiratory disease divided by the total number of hospital visits for all diseases. Here, respiratory diseases are defined as diseases corresponding to ICD-9 codes 460–466 and 470–478, which include acute respiratory diseases, upper respiratory infections, upper respiratory tract infections, among others. The detailed information for codes 460–466 and 470–478 is listed in Table 1.

**Table 1:** Diseases corresponding to ICD-9 codes 460–466 and 470–478.

Code	Disease
460	Acute nasopharyngitis (Common cold)
461	Acute sinusitis
462	Acute pharyngitis
463	Acute tonsillitis
464	Acute laryngitis and tracheitis
465	Acute upper respiratory infections of multiple or unspecified site
466	Acute bronchitis and bronchiolitis
470	Deviated nasal septum
471	Nasal polyps
472	Chronic pharyngitis and nasopharyngitis
473	Chronic sinusitis
474	Chronic disease of tonsils and adenoids
475-478	Other diseases of upper respiratory tract

The raw data of PM_2.5_ (unit: *μg*/*m*^3^) are collected from the Taiwanese Central Weather Bureau (https://www.cwb.gov.tw/). The concentrations of PM_2.5_ are recorded in 70 meteorological stations across Taiwan in 2012. Each station recorded the raw PM_2.5_ concentration every hour of every day in 2012. For each station, the recordings throughout the year were averaged. Since 70 meteorological stations do not correspond to the 349 regions, the Kriging technique [13] was further applied to interpolate the PM_2.5_ value for each region.

To better detect the influence of PM_2.5_ on respiratory diseases, we collected the smoking rate and number of hospitals in the 349 regions in Taiwan as control variables. The smoking rate data were obtained from the Adult Smoking Behavior Survey, a survey conducted by the Health Promotion Administration in Taiwan. Here, the smoking rate is defined as the percentage of people over 18 years of age who have previously smoked more than a total of 100 cigarettes and have used tobacco products in the past 30 days. The number of hospitals were collected from the Taiwanese NHIRD for the 349 regions in 2012. Here, the number of hospitals is defined as the total number of clinics, district hospitals, regional hospitals, and medical centers in each region.

### Method

We applied the coherent and generative disease mapping model (CG model) [12] to investigate the relationship between hospital visit rates for respiratory diseases and PM_2.5_. In the past literature of disease mapping models, most research has focused on relative risks, which required the use of internal standardization to calculate the expected number of observations and thus, made the models incoherent and not generative [11, 14–18]. On the contrary, the CG model replaced relative risk with disease incidence, and thus, behaved incoherently and generatively. Consequently, it achieved tighter credible intervals. Thus, in the present work, the CG model was used to estimate the hospital visit rates for respiratory diseases.

To better detect the influence of PM_2.5_ on respiratory diseases, we considered two covariates, namely, the smoking rate and number of hospitals, as control variables. The existing literature has shown that the smoking rate and number of hospitals could influence hospital visit rates for respiratory diseases. A meta-analysis of longitudinal studies, including 216 articles from 1985 to 2013, showed that there were substantial increases in the risks of lung cancer, COPD, and asthma among adult smokers [3]. In addition, the smoking rate was often used as a covariate in previous studies. For example, the Cox proportional hazards model was used to study the association between air pollution and respiratory mortality in Japan [10], which involved adjusting for smoking status. The number of hospitals in each region, as a representation of a region’s economic status, could also influence the hospital visit rates of diseases [19]. Therefore, we included the smoking rate and number of hospitals in the CG model.

The structure of the CG model used in this study was as follows. Assume there were a total of *I* = 349 regions in Taiwan. For region *i*, *Y*_*i*_ was the number of hospital visits for respiratory diseases and *n*_*i*_ was the total number of hospital visits for all diseases. Accordingly, the hospital visit rate for respiratory diseases in region *i* was defined as *p*_*i*_ = *Y*_*i*_/*n*_*i*_. Then, the CG model was written as

$$\begin{array}{c} \begin{array}{cc} Y_{i}|p_{i} & ∼\text{Possion}(n_{i}p_{i}),\\ \text{Logit}(p_{i}) & =\beta_{0}+\beta_{1}{\text{PM}}_{2.5,i}+\beta_{2}{\text{smoking}}_{i}+\beta_{3}{\text{hospital}}_{i}+\phi_{i}. \end{array} \end{array}$$ (1)

The number of hospital visits for respiratory disease in each region was assumed to follow a Poisson distribution with an expected value of *n*_*i*_ *p*_*i*_. The logit transformation of *p*_*i*_ was then modeled using a linear relationship with the PM_2.5_ concentration, the smoking rate, the number of hospitals, and a spatial random effect *ϕ*_*i*_. To spatially model *ϕ*_*i*_, the classic conditionally autoregressive (CAR) distribution was used as a prior [14, 20]. By applying the CAR distribution, each region’s neighboring values were considered to smooth the local rates.

## Results

### Visualization of different variables

**Fig 1:**
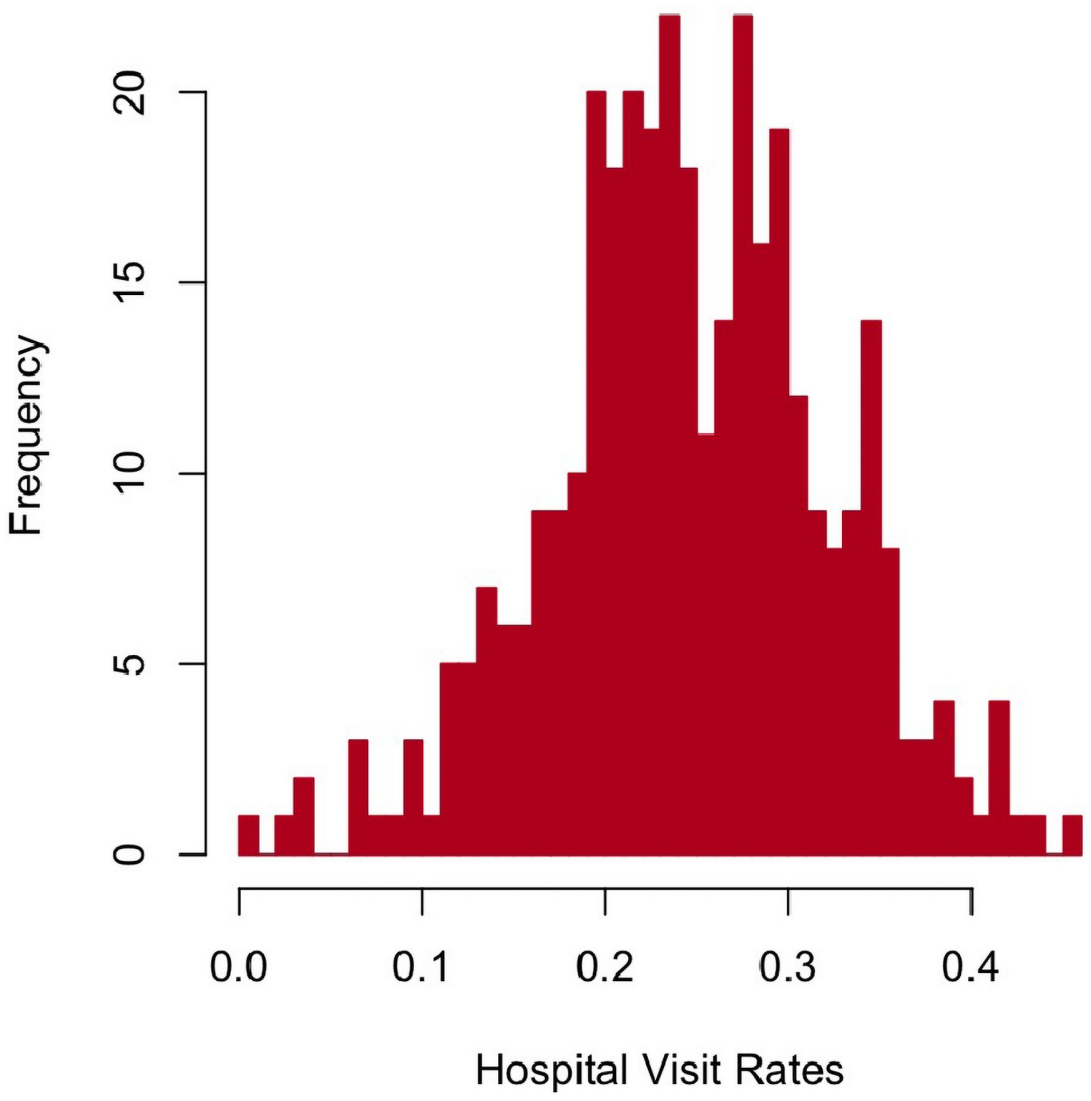
Histogram of hospital visit rates. This figure illustrates the distribution of hospital visit rates for respiratory diseases in 349 third-level administrative regions of Taiwan in 2012. As shown, the hospital visit rates vary in different regions.

Fig 1 showed a histogram of hospital visit rates for respiratory diseases in all 349 regions in Taiwan. It was evident that the hospital visit rates for respiratory diseases in most regions lied in the range of 0.1 to 0.4. The highest hospital visit rate for respiratory diseases was 0.45 for *Shuishang Village*, *Chiayi County*, located in midwestern Taiwan. The lowest hospital visit rate for respiratory diseases was 0 observed in *Dabu Village*, also in *Chiayi County*.

To explore the spatial distributions of PM_2.5_ concentrations, smoking rates, the number of hospitals, and the hospital visit rates for respiratory diseases, we summarized the variable information of 349 third-level administrative regions into 22 second-level administrative regions in Taiwan. Specifically, for each second-level administrative region, we calculated the average values of PM_2.5_ concentrations (after Kriging), smoking rates, hospital numbers, and hospital visit rates for respiratory diseases in all third-level administrative regions under the region’s jurisdiction. Table 2 listed the corresponding results for all 22 second-level administrative regions, as well as their geographical locations in Taiwan.

**Table 2:** Average values of PM_2.5_ concentrations (after Kriging), smoking rates, hospital numbers, and hospital visit rates for respiratory diseases, in 22 second-level administrative regions in Taiwan. The geographical locations of each region in Taiwan are also reported.

Location	County/City Name	PM_2.5_	Smoking Rates	Hospital Numbers	Hospital Visit Rates
North	Keelung City	19.245	0.218	98.429	0.257
North	Taipei City	22.407	0.151	506.083	0.202
North	Taipei County	21.791	0.151	232.069	0.272
Northwest	Miaoli County	25.844	0.211	47.167	0.255
Northwest	Taoyuan County	23.944	0.194	239.462	0.264
Northwest	Hsinchu City	24.473	0.176	243.000	0.271
Northwest	Hsinchu County	23.700	0.197	50.692	0.228
West	Taichung City	31.258	0.197	416.750	0.199
West	Taichung County	29.530	0.197	122.762	0.281
West	Yunlin County	33.067	0.173	62.700	0.255
West	Changhua County	32.804	0.148	69.731	0.251
Center	Nantou County	32.883	0.230	68.769	0.291
Southwest	Chiayi City	35.780	0.171	353.00	0.202
Southwest	Chiayi County	34.303	0.191	30.111	0.27
Southwest	Tainan City	33.534	0.144	280.500	0.238
Southwest	Tainnan Count	33.406	0.144	50.452	0.227
South	Kaohsiung City	40.165	0.187	322.182	0.21
South	Kaohsiung County	36.131	0.187	75.667	0.239
South	Pingtung County	31.856	0.183	40.594	0.249
East	Taitung County	17.927	0.215	23.714	0.242
East	Hualien County	20.014	0.188	40.769	0.22
Northeast	Yilan County	18.720	0.196	48.917	0.222

We first focused on the distribution of PM_2.5_ concentrations. As shown in Table 2, in general, the southwestern and southern regions had relatively higher PM_2.5_ concentrations than other regions. Moreover, the highest PM_2.5_ concentration in 2012 was 40.165 *μg*/*m*^3^ in *Kaohsiung City*, a metropolis located in southern Taiwan. The lowest PM_2.5_ concentration was 13.3 *μg*/*m*^3^ in *Taitung City*, located in eastern Taiwan. The average concentration of PM_2.5_ across 349 regions in Taiwan was 28.9 *μg*/*m*^3^.

Among all 349 third-level administrative regions in Taiwan, the mean of smoking rate was 0.18. As shown in Table 2, the central regions had higher smoking rates than others. Specifically, the highest smoking rate was 0.230 in *Nantou County*, located in central Taiwan, and the lowest smoking rate was 0.144 in *Tainan City* and *Tainan County*, located in southeastern Taiwan. Finally, we investigated the distribution of hospital numbers across 349 regions in Taiwan. Table 2 showed that the number of hospitals varied a lot from region to region. Specifically, *Banqiao City* in *Taipei County* had the largest number of hospitals (1145), while *Daren Village* in *Taitung County*, located in eastern Taiwan, had only 1 hospital.

Table 3 showed the basic statistical summaries of the abovementioned four variables. The coefficient of variation (CV) was used to demonstrate the dispersion of each variable’s frequency distribution. As shown in Table 3, the number of hospitals had the highest CV at 1.52, indicating its scattered characteristics, shown in Table 2. Specifically, the range of hospital numbers lies in the range between 1 to 1145 among all 349 regions in Taiwan. Except for number of hospitals, the coefficients of variation of the other three variables were almost the same and all less than 0.3.

**Table 3:** Main descriptive statistics for hospital visit rates, PM_2.5_ concentrations, smoking rates, and number of hospitals.

	Min	Mean	Max	Standard Deviation	Coefficient of Variation
Hospital Visit Rate	0.00	0.24	0.45	0.07	0.29
PM_2.5_ Concentration	13.3	28.95	40.60	6.71	0.23
Smoking Rate	0.14	0.18	0.23	0.02	0.11
Number of Hospitals	1.00	119.34	1145.00	182.28	1.52

### Correlational relationship between different variables

**Fig 2:**
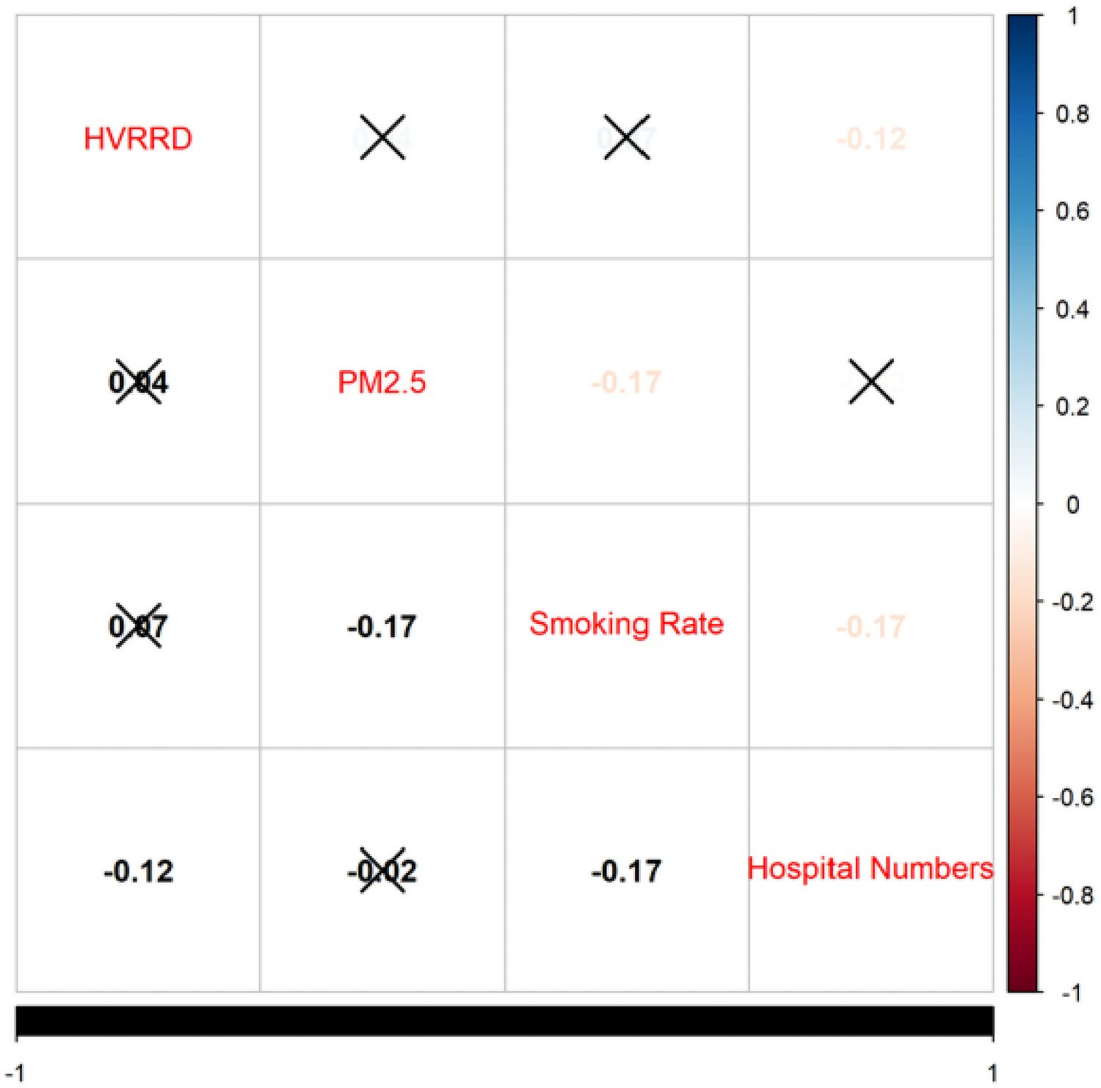
Results of Pearson correlation test for all four variables. HVRRD refers to the hospital visit rate for respiratory diseases. The symbol X indicates that the two variables’ correlation coefficient is not significant with a p-value greater than 0.05.

To explore the relationship among the four variables, Fig 2 showed the Pearson correlation coefficient between different variables. PM_2.5_ had a positive association with the hospital visit rate (0.04), but it was not significant (p-value = 0.5). Moreover, the number of hospitals was significantly negatively associated (-0.12) with the hospital visit rate (p-value = 0.02), but the smoking rate showed no significant association (0.07) with the hospital visit rate (p-value = 0.16).

**Fig 3:**
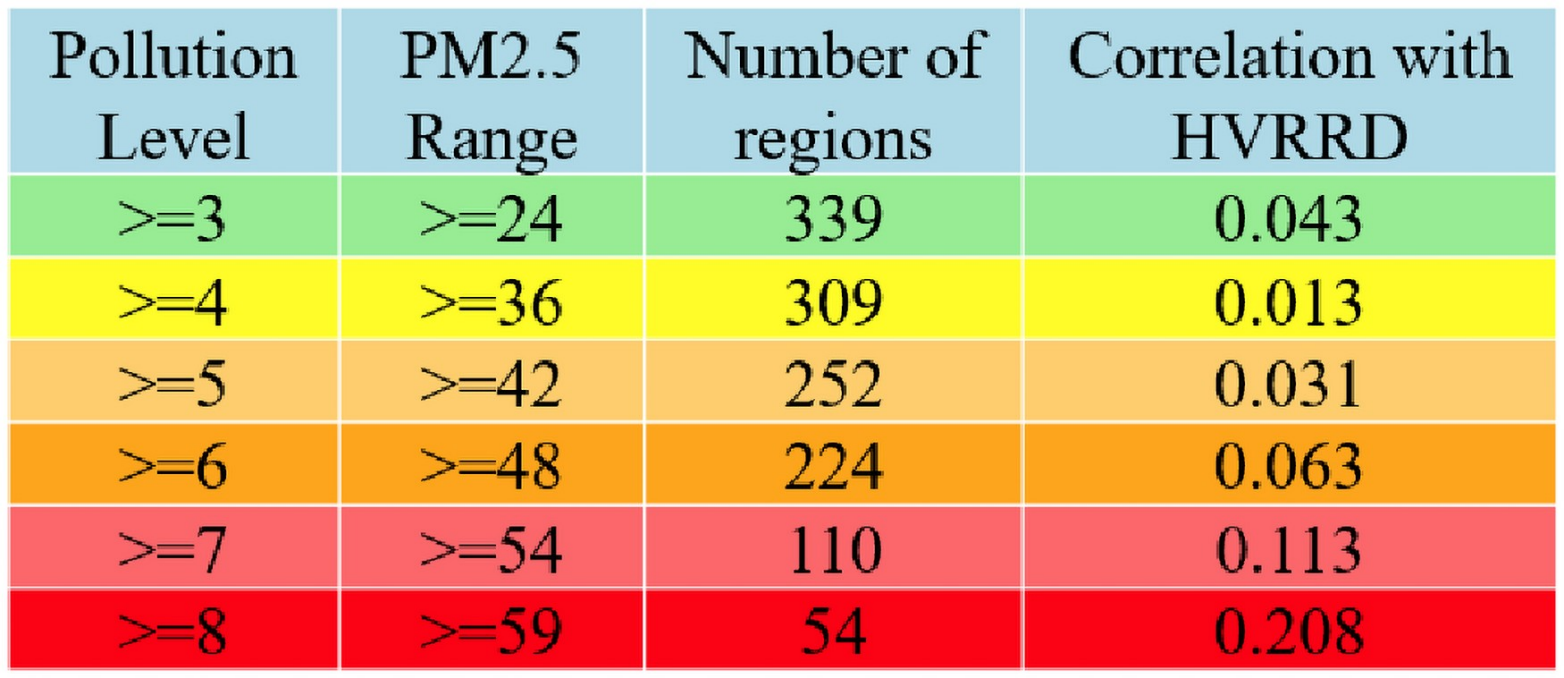
Correlations between hospital visit rates and PM_2.5_ under different pollution levels.

To further explore the relationship between PM_2.5_ and the hospital visit rates for respiratory diseases, regions with PM_2.5_ bigger than a threshold were selected to calculate their correlations with the corresponding hospital visit rates. The thresholds were selected according to the PM_2.5_ pollution levels set by Taiwan. The corresponding results were shown in Fig 3. It was obvious that, as PM_2.5_ increased, its correlation with hospital visit rate also became larger. When the pollution level of PM_2.5_ was bigger than 8, the correlation reached 0.208.

### Modeling results

The CG model was then applied to estimate the hospital visit rates of all respiratory diseases corresponding to ICD-9 codes 460–466 and 470–478. The model results were shown in Table 4. As shown, the mean estimated coefficients for PM_2.5_ concentrations, the smoking rate and the number of hospitals were all positive. The corresponding 95% credible intervals for the three covariates were all larger than zero. These results suggested that, the three covariates all had significant positive effects on the hospital visit rate for respiratory diseases.

**Table 4:** Estimation results of the CG model for the hospital visit rate for respiratory diseases. The estimated mean and 95% credible intervals for different variables are reported.

	Estimated Mean	Estimated 95% Credible Interval
Intercept	-0.60	(-0.89, -0.22)
PM_2.5_ Concentrations	0.84	(0.76, 0.94)
Smoking Rate	2.11	(2.03, 2.22)
Number of Hospitals	3.96	(3.38, 4.43)

We then investigated the influences of PM_2.5_ on the hospital visit rates related to specific respiratory diseases. To this end, the top two diseases with the highest hospital visit rates in 2012 were considered as examples. They were *acute upper respiratory infections of multiple or unspecified sites* and *acute bronchitis and bronchiolitis*, corresponding to ICD-9 codes 465 and 466, respectively. The CG models were then applied separately to the hospital visit rates for these two diseases, and the results were shown in Table 5. In general, the modeling results for these two diseases were in accordance with those for the hospital visit rates of all respiratory diseases in Table 4. As shown, for either disease, the PM_2.5_ concentration, number of hospitals, and smoking rate all showed significantly positive relationships with its hospital visit rate.

**Table 5:** Estimation results of the CG model for *acute upper respiratory infections of multiple or unspecified site* (ICD-9 code 465) and *acute bronchitis and bronchiolitis* (ICD-9 code 466). The estimated mean and 95% credible intervals for different variables are reported.

		Estimated Mean	Estimated 95% Credible Interval
ICD-9 code 465	Intercept	-0.41	(-0.70, -0.17)
	PM_2.5_ Concentrations	0.83	(0.77, 0.89)
	Smoking Rate	1.73	(1.65, 1.81)
	Number of Hospitals	2.55	(1.96, 3.18)
ICD-9 code 466	Intercept	1.39	(1.11, 1.62)
	PM_2.5_ Concentrations	0.80	(0.74, 0.88)
	Smoking Rate	2.14	(2.01, 2.22)
	Number of Hospitals	4.23	(3.56, 4.68)

## Discussion

### Summary of findings

In this work, we aimed to investigate the relationship between PM_2.5_ and hospital visit rates for respiratory diseases in Taiwan. Although the PM_2.5_ concentrations in Taiwan were far below the WHO standard, we still found that different regions in Taiwan had quite different PM_2.5_ concentrations. Therefore, it was still of great importance to explore the influence of PM_2.5_ concentrations on hospital visit rates for respiratory diseases. To this end, we first calculated the Pearson correlation coefficient between PM_2.5_ concentrations and hospital visit rates for respiratory diseases, which was positive, but not significant. Then, the Pearson correlation coefficients for the two variables under different pollution levels were calculated. The corresponding results indicated that relationship between PM_2.5_ and hospital visit rates for respiratory diseases became stronger at higher levels of PM_2.5_. However, it should be noted that the Pearson correlation could only test the linear correlations between two variables without considering other variables. Therefore, the associations between PM_2.5_ concentrations and the hospital visit rate for respiratory diseases should be further investigated via modelling approach.

To this end, we then applied the CG disease mapping model on the respiratory disease data to investigate the relationship between PM_2.5_ concentrations and hospital visit rates for respiratory diseases. We discussed the results of the CG disease mapping model from the following perspectives.

#### The effect of PM_2.5_ concentrations

By applying the disease mapping model and controlling smoking rates and the number of hospitals in each region, we found that PM_2.5_ concentrations had a significantly positive effect on the hospital visit rates for respiratory diseases. Specifically, every 1 *μg*/*m*^3^ increase in PM_2.5_ concentrations would cause a 1.316 (i.e., *e*^0.84^ − 1 = 1.316) increase in the odds ratio of hospital visit rates for respiratory diseases while controlling other variables.

#### The effects of smoking rate

By using the CG disease mapping model, a significant positive effect was observed for the smoking rate. That was, when the smoking rate increased, the hospital visit rate for respiratory diseases would also increase. Specifically, every 1 percent (i.e., 0.01) increase in the smoking rate would cause a 0.072 (i.e., (*e*^2.11^ − 1) × 0.01 = 0.072) increase in the odds ratio of hospital visit rates for respiratory diseases while controlling other variables. This finding for smoking rate was consistent with the existing literature [3, 10, 19].

#### The effect of hospital number

The CG model also detected a significant positive effect from the number of hospitals on the hospital visit rate for respiratory diseases. The positive influence of hospital number might be related to the economic development. When the economy of a region was more developed, it usually had more hospitals. In other words, the hospitals were more accessible to the region’s residents. Therefore, the residents in these regions would be more concerned about their health and more willing to go to hospital than residents in other regions, both of which would lead to the number of hospitals positively influencing the hospital visit rate.

#### The smoothness for hospital visit rates

By considering the spatial effects in the CG disease mapping model, the observed hospital visit rates for respiratory diseases could be smoothed. To illustrate this ideas, we took the region of *Dabu Village* in *Chiayi County* as an example. Specifically, the raw number of hospital visits for respiratory diseases in *Dabu Village* in 2012 was zero. However, this did not mean that the residents in *Dabu Village* were immune to respiratory diseases. In fact, the estimated hospital visit rate for respiratory diseases in *Dabu Village* was 0.251, which was smoothed by its neighborhood regions. This finding verified the advantages of using disease mapping models.

#### Results for two specific respiratory diseases

This study further investigated the influences of PM_2.5_ on the hospital visit rates of two specific respiratory diseases, i.e., *acute upper respiratory infections of multiple or unspecified sites* and *acute bronchitis and bronchiolitis*. By using the CG disease mapping model on the respiratory data for the two diseases, similar modeling results were obtained. However, the degree of influence caused by the same variable behaved slightly differently for different diseases. As for PM_2.5_ concentrations, by controlling other variables in the model for disease with ICD-9 code 465, every 1 *μg*/*m*^3^ increase in PM_2.5_ concentrations would increase the odds ratio by 1.293 on average; while in the model for disease with ICD-9 code 466, every 1 *μg*/*m*^3^ increase in PM_2.5_ concentrations would increase the odds ratio by 1.226 on average. Therefore, when compared with the influence of PM_2.5_ on the hospital visit rates of all respiratory diseases, these two diseases were impacted less by PM_2.5_ concentrations.

#### Comparison with the literature

Our results were consistent with those of the existing literature. First, our findings regarding the positive association between PM_2.5_ and respiratory diseases were also found in such places as the United States, Europe, and Japan [7–10]. However, these prior studies mainly focused on disease mortality, rather than hospital admission rates. For example, Zanobetti and Schwartz [8] conducted studies in the United States, and found that respiratory deaths could increase by 1.68% for every 10 *μg*/*m*^3^ increase in 2-day averaged PM_2.5_ concentrations. In Taiwan, similar results were obtained by some previous studies. For example, Liwei Lai [21], focusing on the health risk of PM_2.5_ in *Kaoping* region in Taiwan, found that, after controlling seasonal and time effects, the monthly trend of respiratory hospital admissions was moderately related to monthly averaged PM_2.5_ concentrations. Another study was conducted by Tsai et al. [22], who aimed to detect the influence of PM_2.5_ on hospital admissions for respiratory diseases in Taiwan. Using a case-crossover approach for data in 2006 to 2010, they found that hospital admissions of respiratory diseases were positively associated with PM_2.5_ levels. However, different from our definition for respiratory diseases, Tsai et al. [22] studied only three kinds of respiratory diseases, that was, pneumonia, asthma, and COPD.

### Limitations

There were several limitations in our study. First, as pointed out in Liwei Lai [21], hospital admissions included only residents who had health insurance and went to clinics or hospitals for medical treatment. However, there were also people who had symptoms but did not go to clinics or hospitals, leading to a missing data problem. Second, we investigated only the effect of PM_2.5_ on respiratory diseases using data collected in one particular year (i.e., 2012), and found a significant positive influence. In the future, data with a longer time span should be analyzed to verify whether this conclusion holds over time. Third, owing to limited data sources, we employed only two covariates, namely, smoking rate and number of hospitals, as controlled variables. In further studies, more covariates, such as PM_10_ and NO_2_, could be included in the disease mapping model to obtain more reliable results. Finally, the relationship between PM_2.5_ and hospital visit rates for respiratory diseases was investigated on a yearly accumulated level. However, when longitude data were available, some spatiotemporal disease mapping models can be applied to extract both spatial effects and temporal effects.

In conclusion, a significantly positive effect caused by PM_2.5_ concentrations was found for hospital admissions of respiratory diseases by using a disease mapping model. Suggested by this work, the harm of PM_2.5_ on respiratory diseases was vividly shown in Taiwan. It could be regarded as a reminder to the whole society that immediate actions should be taken to deal with the air pollution of PM_2.5_ in Taiwan.

## References

[1] Feldman C, Richards G. Appropriate antibiotic management of bacterial lower respiratory tract infections. F1000 Res. 2018. 10.12688/f1000research.14226.1 30079235 PMC6058472

[2] National Center for Biotechnology Information (US). Genes and Disease [Internet]. Bethesda (MD): National Center for Biotechnology Information (US). Respir Dis. 1998. Available from: https://www.ncbi.nlm.nih.gov/books/NBK22167/.

[3] Jayes L, Haslam PL, Gratziou CG, Jimenez-Ruiz C, Leonardi-Bee J. SmokeHaz: systematic reviews and meta-analyses of the effects of smoking on respiratory health. Chest. 2016; 150(1): 164–179. 10.1016/j.chest.2016.03.060 27102185

[4] Jiang XQ, Mei XD, Feng D. Air pollution and chronic airway diseases: what should people know and do? J Thorac Dis. 2016; 8(1): E31. 10.3978/j.issn.2072-1439.2015.11.50 26904251 PMC4740163

[5] Ambient air pollution—a major threat to health and climate. World Health Organization Report. Available from: https://www.who.int/airpollution/ambient/en/.

[6] Xing YF, Xu YH, Shi MH, Lian YX. The impact of PM2.5 on the human respiratory system. J Thorac Dis. 2016; 8(1): 69–74. 10.3978/j.issn.2072-1439.2016.01.19 26904255 PMC4740125

[7] Pope CA III, Burnett RT, Thun MJ, Calle EE, Krewski D, Ito K, et al. Lung cancer, cardiopulmonary mortality, and long-term exposure to fine particulate air pollution. J Am Med Assoc. 2002; 287(9): 1132–1141. 10.1001/jama.287.9.1132 PMC4037163 11879110

[8] Zanobetti A, Schwartz J. The effect of fine and coarse particulate air pollution on mortality: a national analysis. Environ Health Perspect. 2009; 117(6): 898–903. 10.1289/ehp.0800108 19590680 PMC2702403

[9] Analitis A, Katsouyanni K, Dimakopoulou K, Samoli E, Nikoloupoulos AK, Petasakis Y, et al. Short-term effects of ambient particles on cardiovascular and respiratory mortality. Epidemiol. 2006; 17(2): 230–233. 10.1097/01.ede.0000199439.57655.6b 16477266

[10] Katanoda K, Sobue T, Satoh H, Tajima K, Suzuki T, Nakatsuka H, et al. An association between long-term exposure to ambient air pollution and mortality from lung cancer and respiratory diseases in Japan. J Epidemiol. 2011; 21(2): 132–143. 10.2188/jea.JE20100098 21325732 PMC3899505

[11] Dominici F., Peng R. D., Bell M. L., et al. Fine particulate air pollution and hospital admission for cardiovascular and respiratory diseases. *The Journal of the American Medical Association*, 2006, 295(10): 1127–1134. 10.1001/jama.295.10.1127 16522832 PMC3543154

[12] Wang F, Wang J, Gelfand AE, Li F. Disease mapping with generative models. Am Stat. 2018; 1–11.

[13] Wong DW, Yuan L, Perlin SA. Comparison of spatial interpolation methods for the estimation of air quality data. J Expo Sci Environ Epidemiol. 2004; 14(5): 404. 10.1038/sj.jea.7500338 15361900

[14] Knorr-Held L, Besag J. Modelling risk from a disease in time and space. Stat Med. 1998; 17(18): 2045–2060. 10.1002/(SICI)1097-0258(19980930)17:18<2045::AID-SIM943>3.0.CO;2-P 9789913

[15] Sun D, Tsutakawa RK, Kim H, Zhu H. Spatio-temporal interaction with disease mapping. Stat Med. 2000; 19(15): 2015–2035. 10.1002/1097-0258(20000815)19:15<2015::AID-SIM422>3.0.CO;2-E 10900449

[16] Knorr-Held L, Best NG. A shared component model for detecting joint and selective clustering of two diseases. J R Stat Soc Ser A Stat Soc. 2001; 164(1): 73–85. 10.1111/1467-985X.00187

[17] Gelfand AE, Vounatsou P. Proper multivariate conditional autoregressive models for spatial data analysis. Biostat. 2003; 4(1): 11–15. 10.1093/biostatistics/4.1.11 12925327

[18] Crainiceanu CM, Diggle PJ, Rowlingson B. Bivariate binomial spatial modeling of Loa prevalence in tropical Africa. J Am Stat Assoc. 2008; 103(481): 21–37. 10.1198/016214507000001409

[19] Sahni S., Talwar A., Khanijo S., et al. Socioeconomic status and its relationship to chronic respiratory disease. *Advances in Respiratory Medicine*, 2017, 85(2): 97–108. 10.5603/ARM.2017.0016 28440535

[20] Carlin BP, Gelfand AE, Banerjee S. Hierarchical modeling and analysis for spatial data. Chapman and Hall/CRC; 2014.

[21] Lai L. Public health risks of prolonged fine particle events associated with stagnation and air quality index based on fine particle matter with a diameter <2.5*μ*m in the Kaoping region of Taiwan. Int J Biometeorol. 2016; 60(12): 1907–1917. 10.1007/s00484-016-1177-0 27121467

[22] Tsai S, Chiu H, Liou S, Yang C. Short-term effects of fine particulate air pollution on hospital admissions for respiratory diseases: a case-crossover study in a tropical city. J Toxicol Environ Health Part A. 2014; 77(18): 1091–1101. 10.1080/15287394.2014.922388 25072896

